# The European Nucleotide Archive in 2025

**DOI:** 10.1093/nar/gkaf1295

**Published:** 2025-12-03

**Authors:** David Yuan, Alisha Ahamed, Awais Athar, Rajkumar Devaraj, Dipayan Gupta, Muhammad Haseeb, Maira Ihsan, Eugene Ivanov, Vishnukumar Kadhirvelu, Amnon Khen, Manish Kumar, Ankur Lathi, Isuru Liyanage, Lili Meszaros, Colman O’Cathail, Joana Pauperio, Ruben Paz, Stephane Pesant, Nadim Rahman, Jeena Rajan, Iva Tutis, Marianna Ventouratou, Senthilnathan Vijayaraja, Zahra Waheed, Peter Woollard, Tony Burdett, Guy Cochrane, Ugis Sarkans

**Affiliations:** European Molecular Biology Laboratory, European Bioinformatics Institute, Wellcome Genome Campus, Hinxton, Cambridgeshire, CB10 1SD, United Kingdom; European Molecular Biology Laboratory, European Bioinformatics Institute, Wellcome Genome Campus, Hinxton, Cambridgeshire, CB10 1SD, United Kingdom; European Molecular Biology Laboratory, European Bioinformatics Institute, Wellcome Genome Campus, Hinxton, Cambridgeshire, CB10 1SD, United Kingdom; European Molecular Biology Laboratory, European Bioinformatics Institute, Wellcome Genome Campus, Hinxton, Cambridgeshire, CB10 1SD, United Kingdom; European Molecular Biology Laboratory, European Bioinformatics Institute, Wellcome Genome Campus, Hinxton, Cambridgeshire, CB10 1SD, United Kingdom; European Molecular Biology Laboratory, European Bioinformatics Institute, Wellcome Genome Campus, Hinxton, Cambridgeshire, CB10 1SD, United Kingdom; European Molecular Biology Laboratory, European Bioinformatics Institute, Wellcome Genome Campus, Hinxton, Cambridgeshire, CB10 1SD, United Kingdom; European Molecular Biology Laboratory, European Bioinformatics Institute, Wellcome Genome Campus, Hinxton, Cambridgeshire, CB10 1SD, United Kingdom; European Molecular Biology Laboratory, European Bioinformatics Institute, Wellcome Genome Campus, Hinxton, Cambridgeshire, CB10 1SD, United Kingdom; European Molecular Biology Laboratory, European Bioinformatics Institute, Wellcome Genome Campus, Hinxton, Cambridgeshire, CB10 1SD, United Kingdom; European Molecular Biology Laboratory, European Bioinformatics Institute, Wellcome Genome Campus, Hinxton, Cambridgeshire, CB10 1SD, United Kingdom; European Molecular Biology Laboratory, European Bioinformatics Institute, Wellcome Genome Campus, Hinxton, Cambridgeshire, CB10 1SD, United Kingdom; European Molecular Biology Laboratory, European Bioinformatics Institute, Wellcome Genome Campus, Hinxton, Cambridgeshire, CB10 1SD, United Kingdom; European Molecular Biology Laboratory, European Bioinformatics Institute, Wellcome Genome Campus, Hinxton, Cambridgeshire, CB10 1SD, United Kingdom; European Molecular Biology Laboratory, European Bioinformatics Institute, Wellcome Genome Campus, Hinxton, Cambridgeshire, CB10 1SD, United Kingdom; European Molecular Biology Laboratory, European Bioinformatics Institute, Wellcome Genome Campus, Hinxton, Cambridgeshire, CB10 1SD, United Kingdom; European Molecular Biology Laboratory, European Bioinformatics Institute, Wellcome Genome Campus, Hinxton, Cambridgeshire, CB10 1SD, United Kingdom; European Molecular Biology Laboratory, European Bioinformatics Institute, Wellcome Genome Campus, Hinxton, Cambridgeshire, CB10 1SD, United Kingdom; European Molecular Biology Laboratory, European Bioinformatics Institute, Wellcome Genome Campus, Hinxton, Cambridgeshire, CB10 1SD, United Kingdom; European Molecular Biology Laboratory, European Bioinformatics Institute, Wellcome Genome Campus, Hinxton, Cambridgeshire, CB10 1SD, United Kingdom; European Molecular Biology Laboratory, European Bioinformatics Institute, Wellcome Genome Campus, Hinxton, Cambridgeshire, CB10 1SD, United Kingdom; European Molecular Biology Laboratory, European Bioinformatics Institute, Wellcome Genome Campus, Hinxton, Cambridgeshire, CB10 1SD, United Kingdom; European Molecular Biology Laboratory, European Bioinformatics Institute, Wellcome Genome Campus, Hinxton, Cambridgeshire, CB10 1SD, United Kingdom; European Molecular Biology Laboratory, European Bioinformatics Institute, Wellcome Genome Campus, Hinxton, Cambridgeshire, CB10 1SD, United Kingdom; European Molecular Biology Laboratory, European Bioinformatics Institute, Wellcome Genome Campus, Hinxton, Cambridgeshire, CB10 1SD, United Kingdom; European Molecular Biology Laboratory, European Bioinformatics Institute, Wellcome Genome Campus, Hinxton, Cambridgeshire, CB10 1SD, United Kingdom; European Molecular Biology Laboratory, European Bioinformatics Institute, Wellcome Genome Campus, Hinxton, Cambridgeshire, CB10 1SD, United Kingdom; European Molecular Biology Laboratory, European Bioinformatics Institute, Wellcome Genome Campus, Hinxton, Cambridgeshire, CB10 1SD, United Kingdom

## Abstract

The European Nucleotide Archive (ENA; https://www.ebi.ac.uk/ena), hosted at the European Molecular Biology Laboratory’s European Bioinformatics Institute (EMBL-EBI), remains a global, open-access platform for the submission, archiving, dissemination, and reuse of nucleotide sequence data. In 2025, ENA continues to advance its mission of fostering FAIR (findable, accessible, interoperable, reusable) data principles through innovations in interoperability, scalability, and global engagement, providing infrastructure for a rapidly growing volume of data across diverse domains. This article highlights the key developments in 2025, including the progress of the technical transformation, enhanced support for large-scale biodiversity projects, and the implementation of the International Nucleotide Sequence Database Collaboration Global Participation Initiative. We also discuss infrastructure enhancements to handle exponential data growth and improve user experiences and data discovery.

## Graphical Abstract

**Graphical Abstract:**
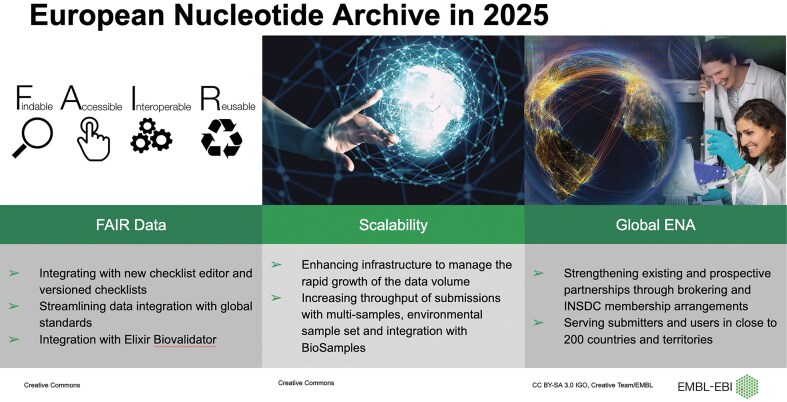

## Introduction

The European Nucleotide Archive (ENA) [1] was established in the early 1980s as a database of records for nucleotide sequences and related information. The ENA has since expanded its mission and today seeks to provide a comprehensive record of the world’s nucleotide sequencing information, covering raw sequencing data, sequence assembly information, and functional annotation. The ENA serves two core functions: it both comprises the globally comprehensive data resource that preserves the world’s public-domain output of sequence data and provides a rich portfolio of tools and services to support the management of sequence data. The open access to data and metadata provided by ENA, and their links to other public databases, serve as a foundation upon which scientific understanding of biological systems may be assembled. The rich portfolio of tools and services provided by the ENA support data coordination activities and partnerships that span the life sciences, covering sequencing-related domains such as livestock genomics, marine biotechnology, biodiversity, pathogen surveillance, and stem cell biology. These partnerships provision technology, create standards, run data analysis and training, support researchers, and advocate for open data across sequencing-related scientific domains.

The ENA is a founding member of the International Nucleotide Sequence Database Collaboration (INSDC) [2], a long-standing global data exchange consortium. With our partners in the National Institutes of Health–National Library of Medicine National Center for Biotechnology (NCBI) [3] in the United States and the Research Organization of Information and Systems DNA DataBank of Japan (DDBJ) [4], the ENA has served as a globally trusted repository for nucleotide sequences, operating in accordance with FAIR (findable, accessible, interoperable, reusable) principles [5] continuously.

In 2025, ENA builds on its legacy of open data advocacy by addressing emerging and ongoing challenges in genomics, such as the increasing volume of high-throughput sequencing data and the need for richer metadata to support cross-disciplinary research.

Focus areas for 2025 have included:

1. *FAIR data* : Integrating with the new checklist editor and versioned checklists to streamline data integration with global standards; validating metadata through integration with Elixir Biovalidator.
2. *Scalability*: Enhancing infrastructure to manage the rapid growth of the data volume and higher throughput of submissions.
3. *Global ENA*: Strengthening existing and prospective partnerships through brokering and INSDC membership arrangements.

## ENA content and services

The ENA provides services for the submission, processing, archiving, and dissemination of nucleotide sequence data. Data are submitted to the ENA through Webin submission services and disseminated through the ENA Browser. ENA services are supported by a helpdesk, and online user documentation and training materials. The entry points for the key services are listed in Table 1.

**Table 1.** Description of ENA services and their entry points

Service	Description	Entry point
Data submission	Tools and guidelines to submit or to update data	https://www.ebi.ac.uk/ena/browser/submit
Data access	Tools and APIs to search, browse, filter, and retrieve data	https://www.ebi.ac.uk/ena/browser/search
User support	Form to contact the ENA helpdesk for support or feedback	https://www.ebi.ac.uk/ena/browser/support
Documentation	Guidelines and tutorials on how to use ENA for data submission and retrieval	https://www.ebi.ac.uk/ena/browser/guides

### Submissions

Over 2800 unique submission accounts from 88 countries have submitted data through ENA services over the 12-month period. The ENA has received over 12 400 studies, 2 million samples, 1.2 million raw read datasets, and 500 000 genome assemblies. The ENA helpdesk supported an average of 222 tickets per month, covering a variety of support topics such as submissions and presentation.

## Content growth

**Figure 1.**
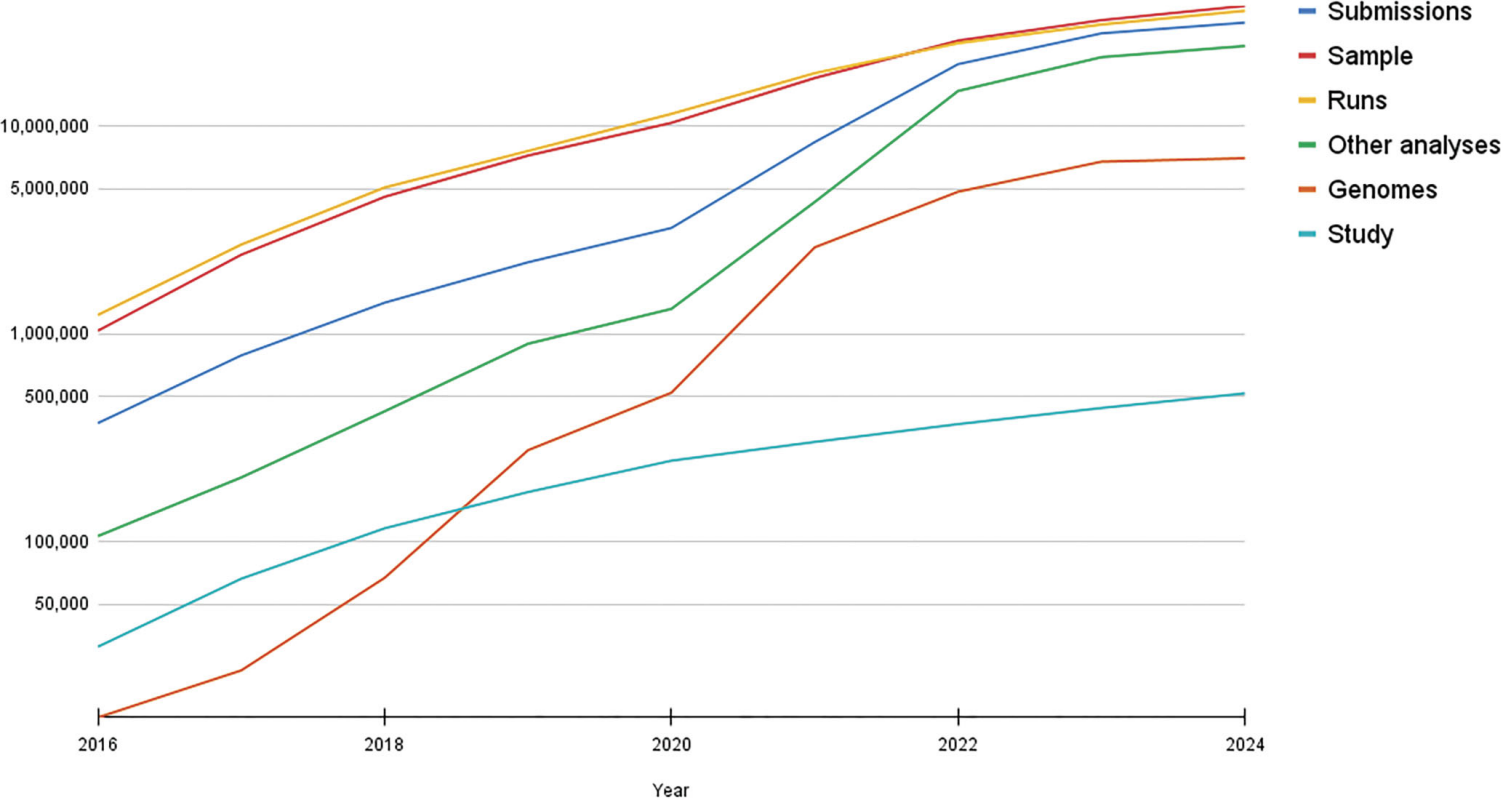
The annual growth of the object counts in all the major data categories in logarithmic scale since 2016. The graph includes the public and private data objects submitted into ENA and archived successfully, excluding the data mirrored from the INSDC partners and withdrawn or replaced records (see https://ena-docs.readthedocs.io/en/latest/faq/release/data-availability-policy.html for definitions). Overview of ENA data type: a *Study* groups related data and controls release. Each *Sample* describes the source material and taxonomy. *Runs* relates to raw sequencing reads submitted. *Genomes* relates to assemblies. *Other analyses* pertains to secondary analysis results derived from sequence reads, not including assemblies, e.g. sequence variation, primary metagenomes. A *Submission* manages actions such as adding, updating, or releasing data.

Continual growth across all ENA submittable objects in 2025 is observed, as shown in Fig. 1. In addition, ENA continues exchanging data and metadata with INSDC partners: DDBJ and NCBI. There are 12 million sequences and 5 million runs in ENA via direct submission and mirroring from NCBI and DDBJ this year. In total, over 37 million raw read datasets are now archived at the ENA, and 26 trillion nucleotides’ worth of sequence information is represented in our assembled and annotated sequence collections.

## Dissemination

The ENA Browser is used by 500 000 monthly web visitors on average, while programmatic consumers make 192 million requests to our application programming interfaces (APIs) like the ENA Portal (Advance Search) API for highly customizable search of the ENA metadata (https://www.ebi.ac.uk/ ena/portal/api) and the ENA Browser API for downloading sequence data and metadata summary (https://www.ebi. ac.uk/ena/browser/api) each month. On average, the ENA Portal (Advance Search) API receives 28 million requests from 57k unique hosts per month, serving 1.6 terabytes (TB) of metadata, while the ENA Browser API serves over 90 million requests from 62k unique hosts per month, providing 3.3 terabytes (TB) of metadata and data. Moreover, there is a total of 52.22 PB of data downloaded from FTP (83.49% by download counts), Aspera (16.43%), and Globus (0.08%) servers in 12 months, with 73.6% of the generated FASTQ files enabling downstream analysis with high-quality data. ENA plays a critical role in making genomics data FAIR.

## Service enhancements in 2025

Scale remains a central challenge for the ENA. The work over the past year reflects how this challenge is being addressed. The ENA has introduced technical optimizations for data turnaround times, new technology stacks to improve data FAIRness, and improved global outreach.

### Graphical taxonomy view in ENA Browser

#### Graphical taxonomy view in ENA Browser

The ENA Browser taxonomy exploration functionality has been enhanced with a new graphical view, complementing the existing tree view. This enhancement was introduced to ease the exploration and interpretation of large, complex taxonomic trees in the traditional linear format. The graphical view provides a more intuitive and dynamic representation of taxonomic relationships, allowing users to visually identify the connections. It also serves as a pilot implementation for better representing relationships between data, which could be extended in the future to better illustrate the relationships of objects within the ENA overall.

Users can access the graphical taxonomy view directly from any taxonomy page in the ENA Browser by selecting the “Graphical View” option. This interactive visualization allows users to navigate taxonomic hierarchies more intuitively through three display modes:

- *Full Hierarchy*, which expands to show all ancestors and descendants of a selected tax ID.
- *All Children*, which reveals all direct descendants.
- *Minimal View*, which focuses on the immediate parent and a limited subset of children.

**Figure 2.**
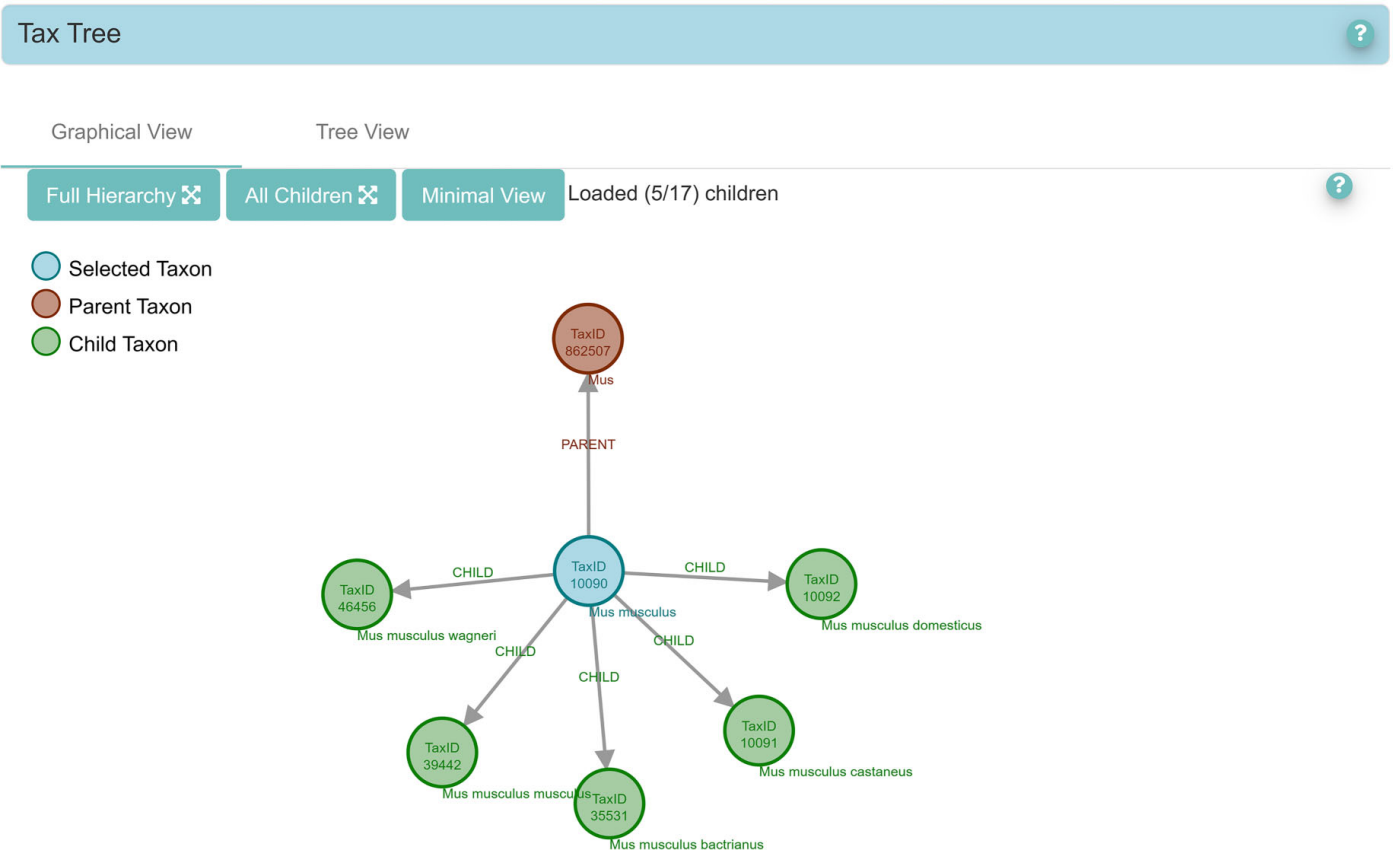
The taxonomy hierarchy of taxon 10090 (Mus musculus—house mouse), showing its parent and the minimal representation of its child taxons.

This feature provides flexible, user-driven navigation of taxonomy data, improving accessibility and comprehension of complex taxonomic relationships (Fig. 2).

### ENA Data Hubs Portal

**Figure 3.**
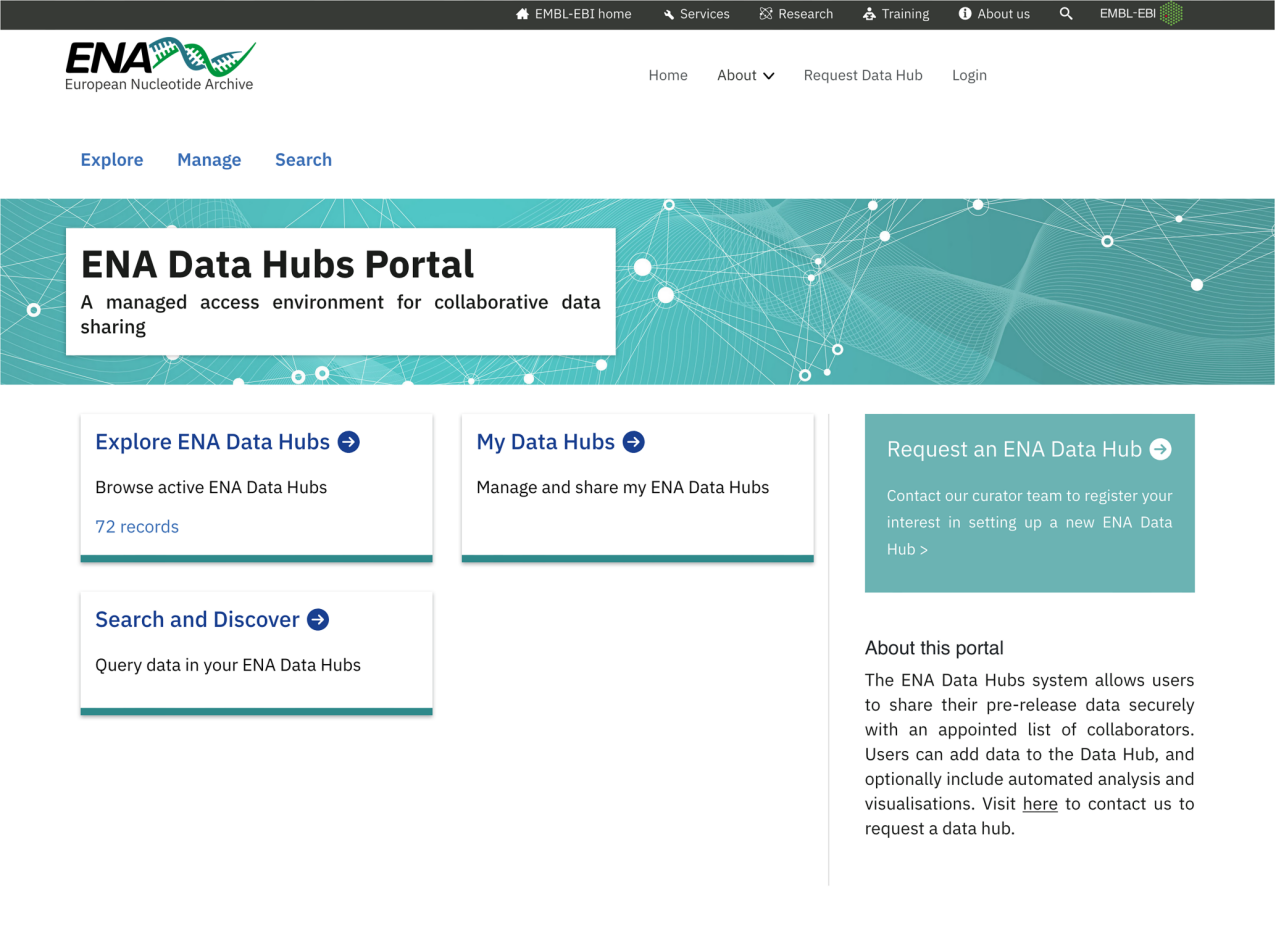
ENA Data Hubs Portal homepage, presenting an interface for the data hubs for registration, setup, and management.

The ENA Data Hubs Portal (Fig. 3) provides an interface for the data hubs at ENA, which are workspaces and sets of tools that enable users to share, analyse, visualize, search, and retrieve their sequence data. The data hubs are built on top of ENA infrastructure (utilizing the same storage, data, and metadata models) and extend on COMPARE data hubs and SARS-CoV-2/pathogen data hubs, which provided specific use-cases for food-borne, antimicrobial resistance, and other infectious disease outbreaks (e.g. SARS-CoV-2). A key feature is the ability for a group of users to collaborate on data, sharing it amongst themselves initially whilst preparing their scholarly publication and coincident public data release (in line with ENA’s data release policy). This differs from ENA’s more-personalized Webin accounts for users. Overall, this supports collaborative researchers and groups to better structure, manage, and prepare their data prior to publication. The portal now supports applications beyond infectious disease, and automated data hub setup and management.

## Selected developments in 2025

### FAIR data advancements

#### Checklist editor & JSON schema management

A new checklist editor has been successfully developed and rolled out in 2025, replacing the legacy editor. The primary goal of this rollout is to modernize the checklist management by transitioning to a JSON-based system that is easier to maintain and extend. New checklists are created using the new checklist editor and stored in a centralized JSON schema store application accessible here. The new checklist editor also supports versioning of checklists and ensures backward compatibility, allowing older submissions to remain valid even as checklists evolve. When a sample needs to be validated, the corresponding checklist is retrieved from the schema store, and both the sample metadata and checklist schema are passed to the Biovalidator for validation. This integrated flow ensures consistent structure, improved data quality, and seamless interoperability with existing services, marking a significant step toward a more sustainable and flexible metadata infrastructure.

Improved standardization and interoperability: checklist versioning allows non-backwards-compatible changes so that we can easily adapt to updates to standards, e.g. Genome Standard Consortium (GSC)-based checklists and field names [6]. The Biovalidator provides transparent JSON schema-based validation. These schemas can be obtained from the JSON schema store API. We continue to work closely with standards organizations to facilitate standardization and interoperability, including GSC, GA4GH [7], and TDWG [8], e.g. in environmental DNA [9] and ancient DNA [10].

#### Submission of environmental sequence set

Submissions of sequences (Amplicon Sequence Variants, ASVs) derived from targeted environmental DNA sequencing projects of a single locus from multiple samples/organisms, with associated annotations (ASV tables), are now enabled. It allows flexible submissions, by decoupling taxonomic annotations from the sequence to sample mapping, and therefore enabling taxonomic reannotations where needed. This development bridges a gap in structured environmental DNA data publication and follows from previous and ongoing community efforts towards FAIR eDNA metabarcoding data sharing (e.g. BiCIKL, BGE, and FAIRe).

#### Enhanced Webin-CLI functionality

Webin-CLI now supports multiple sample submissions and batch submissions, enabling users to submit several samples in a single command or as part of a larger batch process. This enhancement significantly improves throughput, as it reduces the need for repeated, single-sample submissions and minimizes manual intervention. It also streamlines automation workflows, allowing researchers, particularly those working with large-scale metagenomic datasets, to integrate Webin-CLI into their existing pipelines for high-volume data uploads. By processing multiple submissions at once, users can achieve faster turnaround times, more consistent metadata handling, and better scalability for projects involving thousands of samples.

### Foundational enhancement for FAIR data

#### Higher throughput of CRAM reference registry

ENA is hosting the public CRAM Reference Registry (https://www.ebi.ac.uk/ena/cram/swagger-ui/index.html), implementing the GA4GH RefGet standard [11] to provide reference sequences silently and automatically when CRAM files are parsed by informatics tools. This registry used to suffer throughput issues, and tools like Samtools took longer to read the CRAM files or even reported time-out errors. We improved the network architecture and caching strategy, improving the throughput 169-fold. We are migrating the registry to a database backend better suited for the registry. We are expecting the migration to further improve the performance and throughput of the CRAM Reference Registry.

#### Integration of ENA workflows with BioSamples

The integration of the ENA workflows with the BioSamples database [12] is a major milestone in sample data management. This collaboration involved transferring ENA’s entire sample management workflow, including validation, storage, accessioning, and metadata exchange with INSDC partners, to BioSamples. By delegating these responsibilities, ENA is able to reduce operational overhead while leveraging BioSamples’ specialized infrastructure and centralized validation pipelines. This transition leads to consistent, non-redundant, higher-quality sample metadata across the system.

This workflow integration significantly streamlines the submission process for users, with BioSample registration occurring seamlessly in the backend, not requiring changes to the submission process from the user point of view. This has introduced a clearer separation of responsibilities: ENA continues to focus on sequence data, while BioSamples serves as the authoritative source for sample metadata. With robust APIs, support for bulk and programmatic submissions, and full INSDC-compliant data exchange, the workflow integration establishes a scalable, sustainable foundation for long-term metadata interoperability between the two archives. More importantly, it creates more avenues for enhancing interoperability of data between repositories with the tighter integration of multi-omics databases centred around BioSamples.

### Global ENA

The ENA has been recognized as an ELIXIR Core Data Resource, a set of European data resources of fundamental importance to the wider life-science community and the long-term preservation of biological data [13]. This is a testament to the importance of ENA to the research communities in Europe. The ENA has also been selected as one of the Global Core Biodata Resources, demonstrating the fundamental importance of the ENA to the users around the globe [14]. The global impact of the ENA has been enhanced by the following efforts throughout the year:

#### Brokering network

The ENA continues to strengthen its network of data submissions brokers—institutions or consortia that do not own the data but instead submit it on behalf of, and attribute credit to, the original data producers or owners. These are often ‘power users’ responsible for sharing high volumes of data whilst maximizing adherence to FAIR data standards. To date, 62 broker accounts have been registered with the ENA across 19 countries, extending to Portugal this year. The Australian Tree of Life project, supported by Australia Biocommons, is a further notable addition to our brokering network.

Brokered submissions now account for 13% of all public raw sequence reads and 21% of assembled sequence data by record counts in ENA—reflecting an almost three fold increase in brokered raw read data compared to the previous year (see Supplementary Figs S1 and S2). To ensure our brokers receive the highest-quality support, we have created a dedicated, high-priority broker helpdesk queue, which has received 100 tickets since its inception last year. The brokers have also helped ENA by submitting data on behalf of consortium partners with better consistency, and in many cases, with automated workflows.

#### Global engagement

ENA team members have continued to work, along with counterparts from INSDC members with organizations around the world to explore opportunities for membership of INSDC, including through technical and policy discussion.

The ENA has worked on expanding engagement and outreach through presence on social media platforms and delivery of training workshops. ENA has been using platforms such as BlueSky and LinkedIn to engage with users and inform them of its tools and services. It has also been involved in 30 training sessions and workshops, both delivered internally as part of EMBL-EBI courses, as well as external training, instructing researchers on data standards, submission, and retrieval.

Finally, ENA team members are actively involved around UN discussions, such as at the World Health Organization and Convention on Biological Diversity, relating to access and benefit sharing, such as by contributing to informal dialogues and support for negotiations.

## Data coordination activities

**Figure 4.**
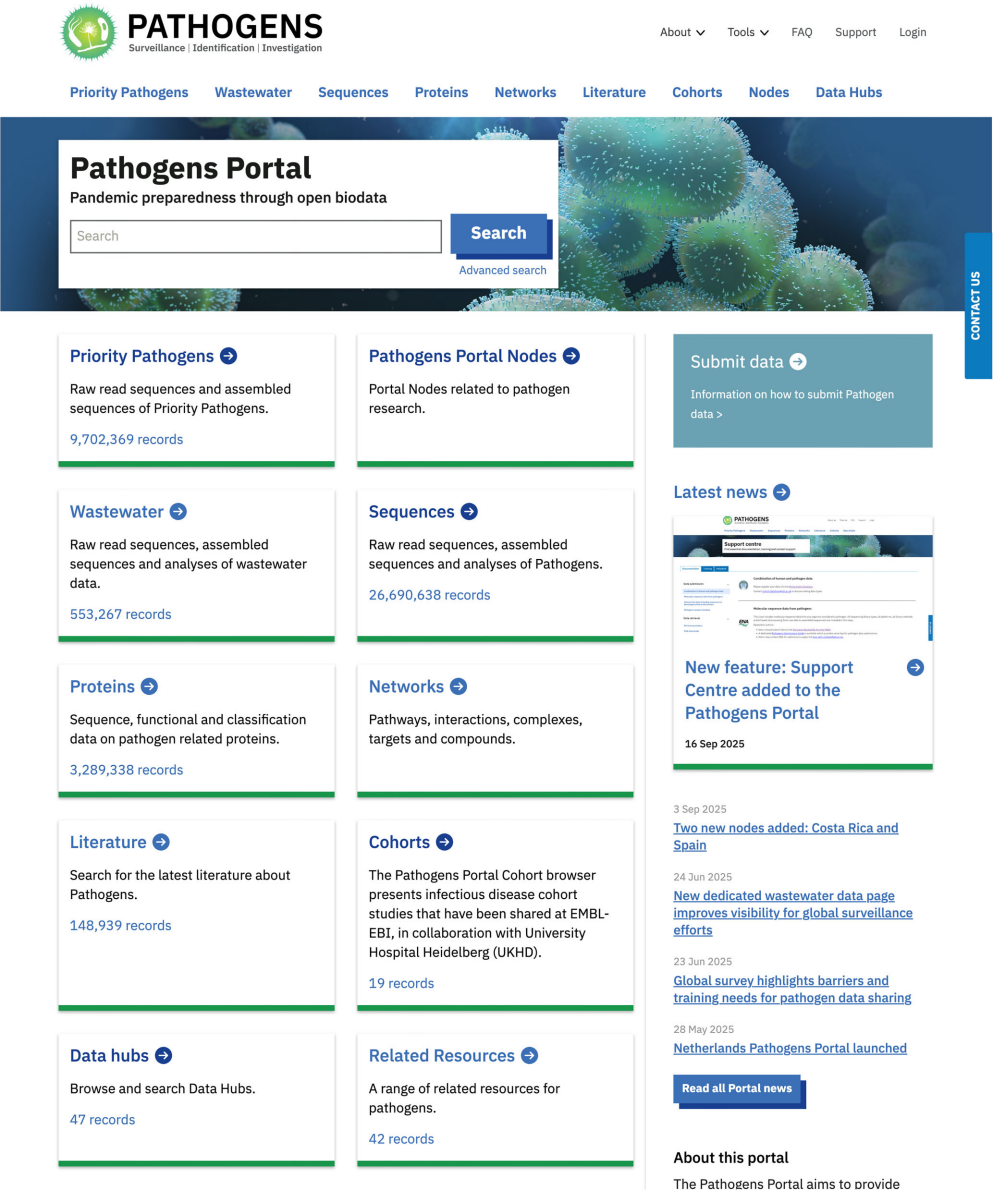
Pathogens Portal homepage, presenting an overview of data that can be discovered on the portal.

During the past year, we have expanded the data management and presentation services for the Pathogens Platform as an example of a resource built on top of ENA and its tools/services. The Pathogens Portal (Fig. 4) was developed with the aim to present a diverse set of multi-omics and multi-modal (including nucleotide sequence) life-science data related to infectious diseases and pathogens. Therefore, it supports researchers, data resources, analysts, public health officials, policymakers, and more within their work with appropriate and structured data and metadata. A full description of the portal is beyond the scope of this publication. It is a tangible example that ENA enables domain-specific data coordination for many scientific communities.

## Future directions

In 2025, ENA stands at the intersection of exponential growth of sequencing output, rapidly evolving metadata standards, and increasing demands for interoperability across research domains. The team will focus its effort on the following:

- Completing the decoupled annotation system by transforming the data format for annotation from EMBL Flatfile to GFF3 [15], which is widely adopted in the genomics community.
- Scaling infrastructure towards the projected 150 PB of data within 5 years. There are two major initiatives planned with minimum impact on user experiences.
  - Migration from SQL database to noSQL databases.
  - Minimizing the presence of certain read formats.
- Indexing ENA data for advanced search incrementally, which will lead to higher performance and shorter overall time from submission to data being accessible in the ENA Browser.

Through sustained investment in technology, standards, and community engagement, the ENA will continue to serve as a cornerstone of global open data infrastructure for the life sciences.

## Supplementary Material

gkaf1295_Supplemental_File

## Data Availability

All data are freely accessible via https://www.ebi.ac.uk/ena. Content is distributed under the EMBL-EBI Terms of Use available at https://www.ebi.ac.uk/about/terms-of-use/.
