# Correction to ‘The European Nucleotide Archive in 2025’

**DOI:** 10.1093/nar/gkag622

**Published:** 2026-06-16

This is a correction to: David Yuan, Alisha Ahamed, Awais Athar, Rajkumar Devaraj, Dipayan Gupta, Muhammad Haseeb, Maira Ihsan, Eugene Ivanov, Vishnukumar Kadhirvelu, Amnon Khen, Manish Kumar, Ankur Lathi, Isuru Liyanage, Lili Meszaros, Colman O’Cathail, Joana Pauperio, Ruben Paz, Stephane Pesant, Nadim Rahman, Jeena Rajan, Iva Tutis, Marianna Ventouratou, Senthilnathan Vijayaraja, Zahra Waheed, Peter Woollard, Tony Burdett, Guy Cochrane, Ugis Sarkans, The European Nucleotide Archive in 2025, *Nucleic Acids Research*, Volume 54, Issue D1, 6 January 2026, Pages D120–D127, https://doi.org/10.1093/nar/gkaf1295

In the originally published version of this manuscript, the forenames and surnames of all authors were erroneously transposed.

The author list has been corrected to read: “David Yuan, Alisha Ahamed, Awais Athar, Rajkumar Devaraj, Dipayan Gupta, Muhammad Haseeb, Maira Ihsan, Eugene Ivanov, Vishnukumar Kadhirvelu, Amnon Khen, Manish Kumar, Ankur Lathi, Isuru Liyanage, Lili Meszaros, Colman O’Cathail, Joana Pauperio, Ruben Paz, Stephane Pesant, Nadim Rahman, Jeena Rajan, Iva Tutis, Marianna Ventouratou, Senthilnathan Vijayaraja, Zahra Waheed, Peter Woollard, Tony Burdett, Guy Cochrane, Ugis Sarkans”.

Additionally, in the ‘author contributions’ section, the corresponding author’s name has been corrected to read: “David Yuan”.

