# Supplementary material for: The European Nucleotide Archive in 2025
Source: Nucleic Acids Res. 2025 Dec 3;54(D1):D120–7. doi: 10.1093/nar/gkaf1295 (PMC12807680; doi:10.1093/nar/gkaf1295)
Supplement: gkaf1295_Supplemental_File [file gkaf1295_supplemental_file.pdf]

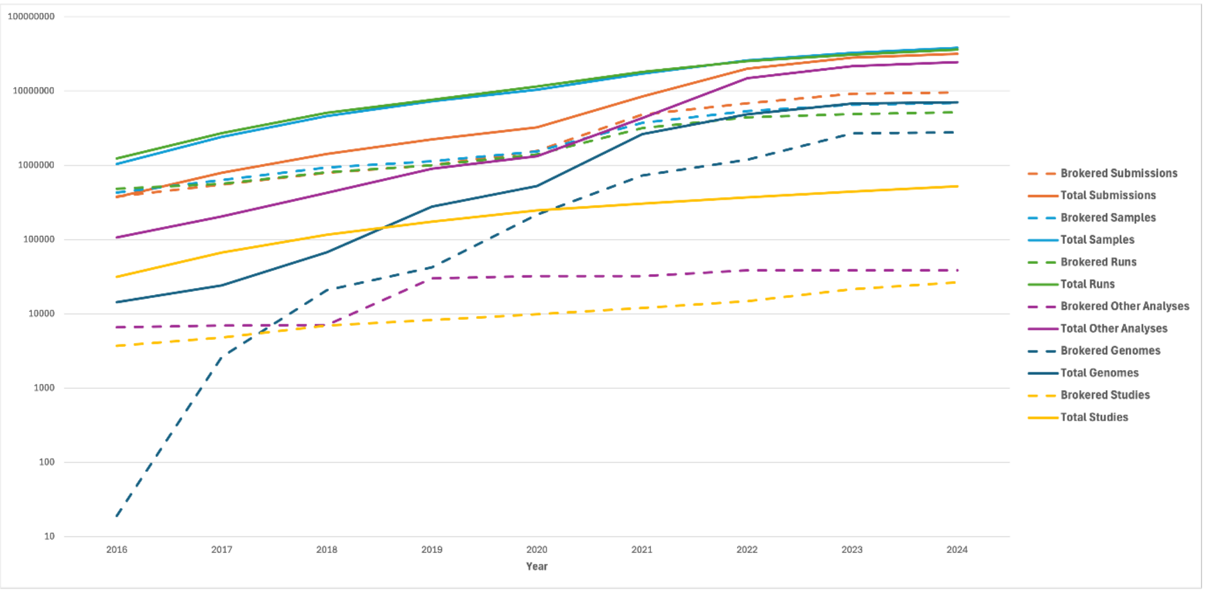

Supplemental Figure 1. Annual growth of object counts in all major data categories in logarithmic scale since 2016, including total objects and broker-submitted objects. A Study groups related data and controls release. Each sample describes the source material and taxonomy. Runs relate to raw sequencing reads submitted. Genomes relate to assemblies. Other analyses pertain to secondary analysis results derived from sequence reads, not including assemblies e.g. sequence variation, primary metagenomes. A Submission manages actions such as adding, updating, or releasing data.

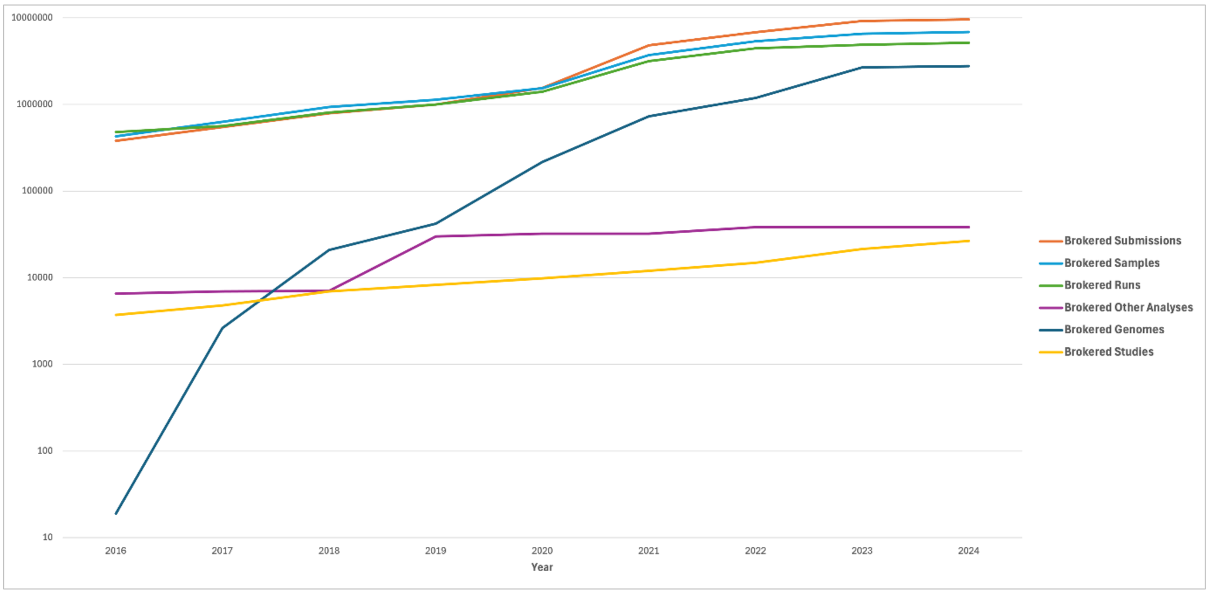

Supplemental Figure 2. Annual growth of broker-submitted object counts in all major data categories in logarithmic scale since 2016. A Study groups related data and controls release. Each sample describes the source material and taxonomy. Runs relate to raw sequencing reads submitted. Genomes relate to assemblies. Other analyses pertain to secondary analysis results derived from sequence reads, not including assemblies e.g. sequence variation, primary metagenomes. A Submission manages actions such as adding, updating, or releasing data.
